# Proteomic profiling of the retinal dysplasia and degeneration chick retina

**Published:** 2010-01-11

**Authors:** Sorcha Finnegan, Joanne Robson, Paul M. Hocking, Manir Ali, Chris F. Inglehearn, Alan Stitt, William J. Curry

**Affiliations:** 1 Centre for Vision Sciences, Institute of Clinical Science, Queen’s University of Belfast, Belfast, Northern Ireland, UK; 2 School of Biological and Biomedical Sciences, Durham University, Durham, UK; 3 Division of, Genetics and Genomics, The Roslin Institute and Royal - School of Veterinary Studies, The University of Edinburgh, Edinburgh, UK; 4 Molecular Medicine Unit, School of Medicine, University of Leeds, Clinical Sciences Building, St. James's University Hospital, Leeds, UK

## Abstract

**Purpose:**

In our previous paper we undertook proteomic analysis of the normal developing chick retina to identify proteins that were differentially expressed during retinal development. In the present paper we use the same proteomic approach to analyze the development and onset of degeneration in the retinal dysplasia and degeneration (*rdd*) chick. The pathology displayed by the *rdd* chick resembles that observed in some of the more severe forms of human retinitis pigmentosa.

**Methods:**

Two-dimensional gel electrophoresis (pH 4–7), gel image analysis, and mass spectrometry were used to profile the developing and degenerating retina of the *rdd* and wild-type (wt) chick retina.

**Results:**

Several proteins were identified by mass spectrometry that displayed differential expression between normal and *rdd* retina between embryonic day 12 (E12) and post-hatch day 1 (P1). Secernin 1 displayed the most significant variation in expression between *rdd* and wt retina; this may be due to differential phosphorylation in the *rdd* retina. Secernin 1 has dipeptidase activity and has been demonstrated to play a role in exocytosis; it has been shown to be overexpressed in certain types of cancer and has also been suggested as a potential neurotoxicologically relevant target. Its role in the retina and in particular its differential expression in the degenerate *rdd* retina remains unknown and will require further investigation. Other proteins that were differentially expressed in the *rdd* retina included valosin-containing protein, β-synuclein, stathmin 1, nucleoside diphosphate kinase, histidine triad nucleotide-binding protein, and 40S ribosomal protein S12. These proteins are reported to be involved in several cellular processes, including the ubiquitin proteasome pathway, neuroprotection, metastatic suppression, transcriptional and translational regulation, and regulation of microtubule dynamics.

**Conclusions:**

This proteomic study is the first such investigation of the *rdd* retina and represents a unique data set that has revealed several proteins that are differentially expressed during retinal degeneration in the *rdd* chick. Secernin 1 showed the most significant differences in expression during this degeneration period. Further investigation of the proteins identified may provide insight into the complex events underlying retinal degeneration in this animal model.

## Introduction

Retinitis pigmentosa (RP) is a major cause of blindness affecting approximately one in every 4,000 individuals [1]. RP constitutes a group of genetically heterogeneous hereditary retinal diseases characterized by degeneration of the rod photoreceptors leading to a loss of peripheral vision. This event has a subsequent effect on the viability of the remaining cone cell population, and these cells may also become progressively depleted [2,3].

Even though the apparent genetic causes of many forms of RP have been elucidated, the underlying mechanisms by which most of these mutations manifest themselves remain elusive. This therefore warrants analysis of the disease mechanisms at the level of the effector molecules, proteins. Consequently, knowledge of protein expression and posttranslational modification at the outset of disease will enable a better understanding of the causal pathology, and this knowledge may enable earlier diagnosis and direct the development of new therapeutics [4].

Investigation of human retinal degeneration is often hampered from the slow progression of the disease and the significant difficulties in obtaining tissues that characterize the true progression of the disease process. Given these difficulties animal models of human retinal pathologies have been central to the study of retinogenesis and subsequent degeneration. Animal models offer the possibility to study pathology in vivo; this has provided new insights into human diseases that can be used to direct diagnosis and treatment. In particular, the chick visual system has long been recognized as one of the most valuable tools to study neural development and more recently, retinal pathologies. Several studies have already been undertaken examining the normal retinal proteome in the chicken [5–7].

The completion of the chicken genome sequencing project identified one billion base pairs of DNA (about one-third as many as humans) and 20,000–30,000 genes (similar to the human genome) [8]. The conservation of gene order between chicken and human is similar to that between human and mouse, in spite of the much greater evolutionary separation [9]. It is therefore possible to predict both candidate disease loci and candidate genes by comparison with the human genome [10]. Chicken models of retinal dystrophy include: retinal degeneration (*rd*), a model for one form of Leber congenital amaurosis [11–14], blindness enlarged globe [14], retinopathy globe enlarged [10], a delayed amelanotic strain of chicken [15,16], and retinal dysplasia and degeneration (*rdd*) [17,18].

The *rdd* chicken was first detected in 1979 by Randall and McLachlan [18]; affected birds exhibited limited vision at hatching and were less active than sighted birds. Histological analysis revealed abnormalities in the retina extending from the retinal pigmented epithelium (RPE) through to the inner nuclear layer (INL). Photoreceptor degeneration became obvious around embryonic day 16 (E16) with subsequent progressive photoreceptor loss; at 5 weeks post hatch, their numbers were greatly diminished and the INL was significantly depleted. By sexual maturity at 15 weeks, most birds were blind, and by 6 months there was no response to visual stimuli [17,19]. The pathology displayed by the *rdd* chick resembles that observed in some of the more severe forms of human RP. Genetic analysis of the *rdd* chicken has revealed a null mutation in the *Mpdz/Mupp1* gene (data not shown). In human, Mpdz/Mupp1 is known to interact with CRB1, mutations in which cause recessively inherited human RP and Leber congenital amaurosis (LCA) [20,21]. This animal model may therefore offer valuable insights into the pathogenesis of RP, and the potential involvement of MPDZ mutations in human inherited retinal disease is currently being investigated.

Normal retinal development is characterized by a wave of neurogenesis and synaptogenesis initiating in the central retina, with a lag as it migrates to the peripheral retina. The specific ages that were chosen for this study permitted us to examine *rdd* retina during the early phase of degeneration, a period that corresponds to the major phases of neurogenesis and synaptogenesis in the wt retina. Histology, two-dimensional gel profiling, image analysis, and mass spectrometry (MS) were used to characterize the retinal architecture and to detect protein expression changes underlying retinal development and degeneration in *rdd* versus wt chick.

## Methods

### Whole eye and retinal tissue collection

White leghorn chickens (*Gallus gallus domesticus*; Roslin Institute, Edinburgh, UK), fertilized white leghorn eggs, *rdd* chickens, and fertilized *rdd* eggs (Roslin Institute) were maintained by standard feeding and husbandry procedures under a Home Office License. E12 and E13 chicks were killed by chilling in −80 °C for 15 min; E17 and E19 chicks were killed by decapitation; and post-hatch day 1 (P1) chicks were killed by cervical dislocation. The eyes were dissected and retinas for proteomic analysis were carefully peeled free of RPE. Retinas were washed in chilled PBS (137 mM NaCl, 2.7 mM KCl, 4.3 mM Na_2_HPO_4_, 1.47 mM KH_2_PO_4_, pH 7.4) to remove any extraneous RPE, snap frozen in liquid nitrogen within 10 min of death, and subsequently stored at −80 °C. All animal procedures were performed in compliance with the UK Animals (Scientific Procedures) Act 1986.

### Histology

Resected eyes were perforated and immersion fixed in buffered 4% (w/v) paraformaldehyde (4 h, 4 °C; Sigma-Aldrich, Poole, UK), dehydrated sequentially in 70% (v/v; 2×30 min), 95% (v/v; 2x30 min), 100% (2×30 min) industrial grade methylated spirits followed by immersion in 100% ethanol (2×30 min) and 100% xylene (2×30 min to render the tissue miscible with wax impregnation, embedded, and microtome sectioned (5 μm). Retinal sections were stained with hematoxylin and eosin.

### Retinal protein extraction and 2 dimensional gel electrophoresis (2DE)

A total of three wt and *rdd* chicks were analyzed at E12, E13, E17, E19, and P1. The retinas from each chick were pooled, and retinal protein was collected and processed as previously described [5]. The retinal proteins were analyzed using two-dimensional PAGE (2D PAGE), as previously described [5]. Briefly, retinal tissues were ground to a powder in liquid nitrogen, and protein was extracted in 40 mM ammonium bicarbonate buffer to which dithiothreitol (DTT; Melford Labs, Suffolk, UK) was added (approximately 5:1 [w/v]). The sample was sonicated (15 min, 4 °C), extracted for 1 h on ice with vortexing, dialyzed (24 h, 4 °C) using Slide-A-Lyzer® dialysis cassettes (3,500 molecular-weight cutoff; Pierce, Leicestershire, UK), lyophilized, and reconstituted in lysis solution (7 M urea, 2 M thiourea, 2% 3-[(3-Cholamidopropyl)dimethylammonio]-1-propanesulfonate [CHAPS], 20mM DTT, 0.5% immobilized pH gradient [IPG] buffer, pH 4–7). Protein quantification was performed using a PlusOne 2-D Quant Kit (Amersham Biosciences, Buckinghamshire, UK). Retinal protein extract (1 mg) was diluted to generate a final volume of 400 µl with rehydration buffer (7 M urea, 2 M thiourea, 2% CHAPS, 80 mM DTT, 0.5% IPG buffer containing 3′, 3′’, 5′, 5′’ tetrabromophenolsulfonephthalein (bromophenol blue; Sigma-Aldrich) and added to 18-cm pH 4–7 IPG strips (Amersham Biosciences). Following rehydration (18 h, room temp), isoelectric focusing (IEF) was performed using a Multiphor II system (20 °C) with a MultiTemp III circulator and an EPS 3501 XL power supply (200 V for 1 V hour [Vh], 3,500 V for 3,000 Vh, and held at 35,000 V for 52,000 Vh; Amersham Biosciences).

IPG strips were equilibrated for 15 min in 10 ml equilibration buffer (50 mM Tris-Cl, pH 8.8, 6 M urea, 30% [v/v] glycerol, 2% [w/v] sodium dodecyl sulfate [SDS], bromophenol blue) containing 100 mg DTT. Following this, the IPG strips were added to 10 ml of fresh equilibration buffer containing 250 mg iodoacetamide (Sigma-Aldrich) for a further 15 min. SDS–PAGE was performed using an Ettan^TM^ DALT*six* vertical system (Amersham Biosciences) with a MultiTemp™ III thermostatic circulator (25 °C; Amersham Biosciences). Proteins were resolved by 2D PAGE under 600 V, 400 mA for 30 min; followed by 600 V, 400 mA, 17 W/gel for 4 h. Gels were then fixed, stained with Coomassie brilliant blue G-colloidal (Sigma-Aldrich), and destained using standard procedures. Gels were stored in 25% (v/v) ammonium sulfate (BDH Laboratories, Leicestershire, UK) solution at 4 °C.

### Image analysis

Gels were scanned as 16-bit grayscale tiff image files with a GS-800 calibrated densitometer (Bio-Rad Laboratories Inc., Hercules, CA), and the protein spot intensities were quantified using Progenesis PG220 version 2006 build 2417.2 (Nonlinear Dynamics Ltd., Newcastle upon Tyne, UK). Gels from wt (n=3 independent biologic replicates) and *rdd* (n=3 independent biologic replicates) retinas from E12, 13, 17, 19, and P1 were imported into five separate experiments, spots were detected and matched, and volumes normalized within triplicate (n=3) gel sets to create an average gel for each age and phenotype. The following parameters for protein spot detection were used: minimum spot radius of 16 (the smallest area perceived as a spot), minimum spot intensity (volume above base level), and split factor 7 (a value between 1 and 9 controls automatic splitting of spots that may have merged together; as this number increases splitting becomes more vigorous). Manual verification and editing of spot matching were performed, and only spots matched in at least two-thirds of the gel images were considered for further analysis. Gels from each experiment were processed using the normalization mode (this erases intensity differences of similar spots in different gels not due to regulation but to experimental variability of the method, e.g., protein load or Coomassie stain intensity). To normalize spot volume, the volume of a spot is divided by the total volume of the entire image (volume is defined as the optical density [OPD]) and is calculated using the following formula: (the OPD of a spot) × (the area occupied by a spot). Subsequently spots were filtered to find significant differences within certain detection limits; only those filtered spots exceeding an intensity threshold of 1.5 to twofold increase or decrease between wt and *rdd* were subjected to further analysis. The threshold regulation factor for the significance level was set at p≤0.05; spots regulated more than the factor required for significance were further considered as candidate spots and subsequently subjected to manual verification. The two average gels (one wt and one *rdd*) from each of the five separate experiments were imported into one new experiment. Any significant differences in spot intensities that were detected in the previous individual experiments were analyzed at all other ages in the new experiment. A Student *t*-test and Analysis of Variance (ANOVA) were used to analyze differences in spot values, and spots that showed significant variation between wt and *rdd* retina were identified by MS.

### Trypsin digestion and Matrix-assisted laser desorption/ionization–time of flight (MALDI-TOF) analysis

Calibration of the mass spectrometer was first performed using Angiotensin I - 1296.6853, adrenocorticotropin (ACTH) [1–17] 2093.087, ACTH [18–33] 2465.199, and ACTH III [7–33] 3657.929. The calibration standard mix must match at least three of these peaks at a max outlier of 50 ppm. Following this calibration samples were internally calibrated using trypsin autolysis peaks at masses 842.500 and 2211.100, the maximum outlier error of this calibration is 50 ppm. If these peaks were not detected, we deferred to the default calibration, which is routinely updated as above. Proteins analyzed by MALDI-TOF (Durham University, Durham, UK) were tryptically digested using Genomic Solutions ProGest (Genomic Solutions®, Ann Arbor, MI), transferred to a microtiter plate (PE Biosystems Symbiot robot; PE Biosystems, Warrington, UK), lyophilized in a vacuum concentrator, resuspended in 10 µl of 0.1% formic acid, sonicated, and spotted onto a MALDI target plate before MS analysis, using a Voyager DE-STR MALDI-TOF mass spectrometer (PE Biosystems).

### Database searching

Peptide peak lists were generated with MASCOT Wizard and submitted to Mascot (version 2.1) for peptide mass fingerprint database searching of the National Center for Biotechnology Information (NCBI) database. The Mowse scoring algorithm [22] was used to determine the significance of the identity; it indicates if the identity is significant and if it would be expected to occur at random with a frequency of less than 5%. Additional criteria were used to ensure correct protein identifications. These included ensuring the matched peptides were the most abundant peaks in the mass spectrum, the theoretical isoelectric point and molecular weight of the identified protein correlated with the spot’s position on the 2D gel, and sequence coverage was high.

Small-molecular-weight proteins are often difficult to definitively identify because they generate only a small number of short peptide fragments after tryptic digestion. Since Mascot does not have an option to search “*Gallus gallus”* as the taxonomy, “Protein Prospector” was used to match small-molecular-weight proteins. The MS-Fit tool in Protein Prospector offers the option of refining the search parameters by entering the pI of the protein based on its position on the 2D gel and refining the taxonomy to *Gallus gallus*. After scanning the possible matches generated in Protein Prospector, the matched peptides were cross-referenced with the raw spectra. When the matched peptides were the most intense peaks in the spectra, there was a higher possibility that the match was correct. Settings used in Protein Prospector were: Database Search: NCBInr.2006.02.16; MW of Protein: All; Protein pI: 4–7; Min No of peptides required to match: 4; Taxonomy: *Gallus gallus*; Report MOWSE scores: PFactor 0.4; Instrument: MALDI TOF; Peptide masses are: monoisotopic; Mass Tolerance: ±50 ppm; Digest: Trypsin; Max no of missed cleavages: 1; Cys modified by: acrylamide.

## Results

**Figure 1:**
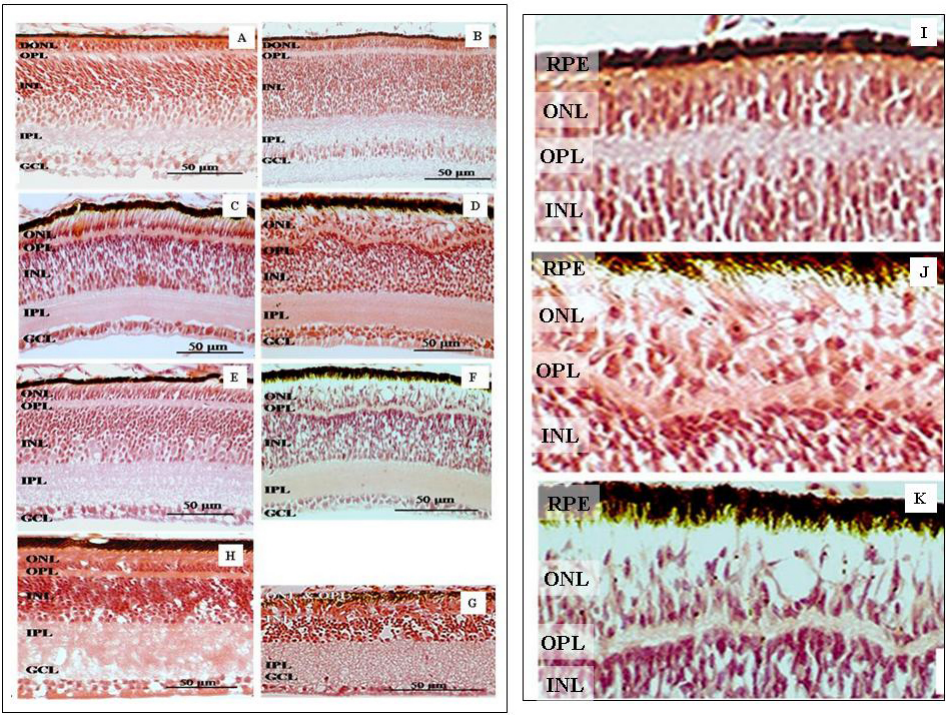
Retinal dysplasia and degeneration (*rdd)* retinal histology. Microtome sections of retina stained with hematoxylin and eosin. Normal retinal morphology is evident in the developing wt chick at embryonic day (E)13 (**A**), E18 (**C**), and post-hatch day (P)1 (**E**). At E13 the gross retinal morphology of the *rdd* retina (**B**) is similar to that of the wt retina; however, the degenerative changes are obvious in the *rdd* retina at E18 (**D**) and P1 (**F**). Magnified images of the outer plexiform layer (OPL) and outer nuclear layer (ONL) show the progressive disorganization of the outer retinal layers of the *rdd* chick from E13 (**I**), E18 (**J**), and P1 (**K**).

Histology was performed to characterize the progression of retinal development and subsequent degeneration in the *rdd* retina with age-matched wt chick retina. This data aided the interpretation of 2D profiles and observed protein expression changes. Histology revealed that the characteristic developing laminated retinal architecture was comparable at E13 (Figure 1A,B) in wt and *rdd* retina. By E18, however, the outer plexiform layer (OPL) and outer nuclear layer (ONL) displayed a degree of disorganization in which the characteristic demarcation between these two layers had started to deform; this in turn led to folding in the ONL and INL (Figure 1C,D). By P1 the photoreceptor layer displayed distinct disorganization. At this stage *rdd* chicks have only limited vision [17] and the pathological changes in the retinal architecture were quite pronounced, with buckling of the OPL and detachment of photoreceptors from the RPE (Figure 1E,F). Magnified images of the ONL and OPL in the *rdd* chick at E13 (Figure 1I), E18 (Figure 1J), and P1 (Figure 1K) clearly demonstrate the degenerative changes occurring in the outer layers of the retina with buckling of the OPL and detachment of the photoreceptors from the RPE. By P21 the *rdd* retina was reduced in thickness to about half that of the wt retina, the INL thickness was greatly diminished, and the OPL, ONL, and outer segments were no longer distinguishable as these layers had atrophied and merged in *rdd* chicks (Figure 1G,H). This verifies the lack of electroretinogram (ERG) response by 3 weeks post hatch [17].

**Figure 2:**
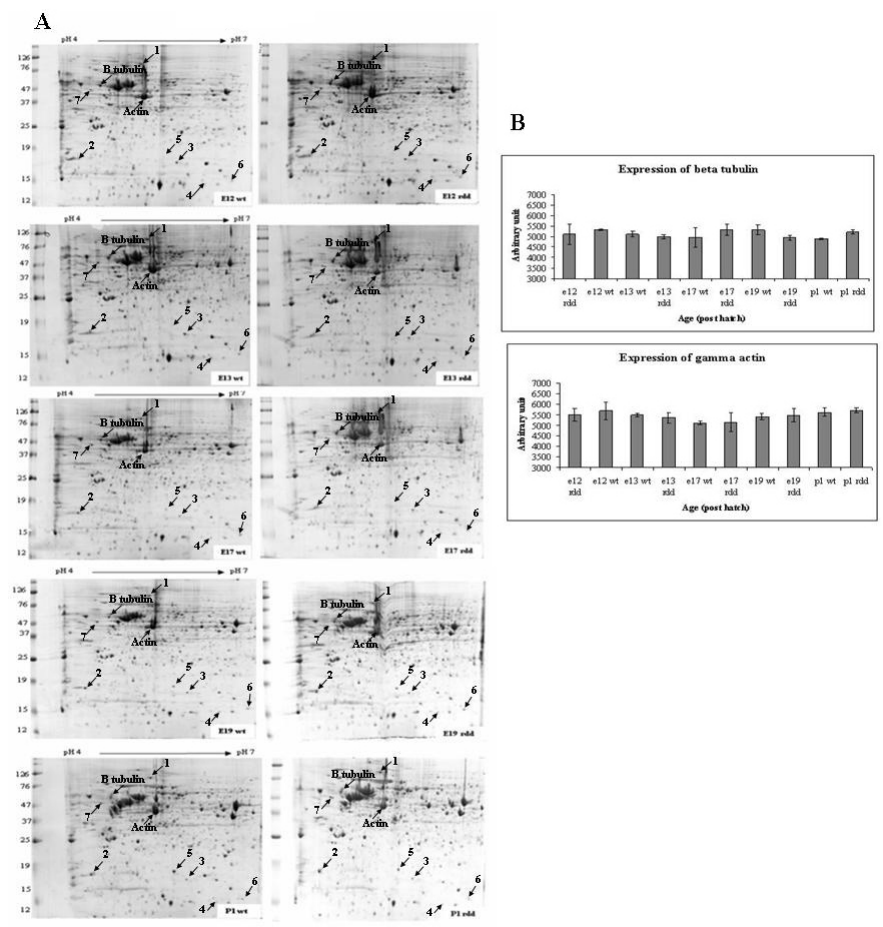
Protein profiles from wild type (wt) and retina dysplasia and degeneration (*rdd*) retina. **A**-**D**: Representative 2D protein profiles from embryonic day (E)12, E13, E17, E19, and post hatch day (P)1 *rdd* and wt chick retina displaying positions of proteins that were identified by MS (Table 1). The positions of two housekeeping proteins (β-tubulin and actin) are also shown. Proteins were extracted using 40 mM ammonium bicarbonate; 1 mg of retinal protein was separated in the first dimension on an 18-cm pH 4–7 IPG strip and in the second dimension on a 12% polyacrylamide gel. The protein spots were visualized with Coomassie brilliant blue G-colloidal. **B**: Levels of actin and tubulin in the *rdd* and wt retina. γ-Actin and β-tubulin were identified by MS and used as loading controls for the gels. There was no significant difference in the levels of actin or tubulin in the wt and *rdd* gels from E12 to P1 (**B**). Bar charts show age plotted against arbitrary units, with SEM.

Proteomic analysis was performed to identify proteins that were differentially expressed in the wt versus *rdd* retinas. An average of 1,531 spots per gel were detected in the pH range 4–7 (Figure 2A), which was chosen for these analyses because it provided the best resolution of the major protein population present in the retinal samples. Using Progenesis image analysis, seven proteins were detected that demonstrated significant variation in expression in the *rdd* versus wt retina. These proteins were identified by MS (Table 1, Figure 2A) and included valosin-containing protein, β-synuclein, stathmin 1, histidine triad nucleotide-binding protein, nucleoside diphosphate kinase, 40S ribosomal protein S12, and secernin 1. γ-Actin and β-tubulin were also identified by MS and used as loading controls for the gels. There was no significant difference in the levels of actin and tubulin in the wt and *rdd* gels from E12 to P1 (Figure 2B). Fold changes in the expression of the identified proteins were calculated (Table 2), probability values were determined using the Student *t*-test, and one-way ANOVA were performed to compare the average normalized volumes of proteins identified by MS from wt and *rdd* retina (Table 3).

**Table 1:** *Gallus gallus* proteins identified by mass spectrometry

**Spot number**	**Protein**	**Accession #**	**Mowse score**	**% Sequence coverage**	***M*_r_ (Da)**	**Calculated pI**	**Number peptides matched**
1	Valosin containing protein (VCP)	gi|113206112**	348	66	89953	5.14	18
2	Synuclein, beta (β-synuclein)		574	44.4	14063	4.4	5
3	Stathmin 1/ oncoprotein 18	gi|50053682 **	97	47	17072	6.18	12
4	PREDICTED: Similar to Histidine triad nucleotide binding protein (Hint-1)		1450	58.7	13759	6.3	9
5	PREDICTED: Similar to Nucleoside diphosphate kinase (NDK1)		355	86.9	17337	5.6	11
6	PREDICTED: Similar to 40S ribosomal protein S12	gi|50742739**	81	75	14935	6.81	8
7	PREDICTED: Similar to KIAA0193/Secernin 1 (SCRN1)	gi|118086002**	106	43	46638	4.69	18

Proteins identified by mass spectrometry that displayed significant differences in expression in the wild type (wt) and retinal dysplasia and degeneration (*rdd*) retina.

**Table 2:** Fold change in protein expression in the retina dysplasia and degeneration (*rdd)* retina relative to wild type (wt) retina (with SEM)

**Protein**	**Fold change of protein expression in *rdd* retina relative to wt at E12**	**Fold change of protein expression the *rdd* retina relative to wt at E13**	**Fold change of protein expression the *rdd* retina relative to wt at E17**	**Fold change of protein expression the *rdd* retina relative to wt at E19**	**Fold change of protein expression the *rdd* retina relative to wt at P1**
VCP	↓ 1.72 (p<0.05)	↓ 2.5 (p<0.05)	↓ 1.8 (p<0.05)	-	-
Β-synuclein	-	↑ 1.5 (p<0.005)	-	↑ 1.85 (p<0.0005)	↑ 1.4 (p<0.05)
Stathmin 1	↓ 1.5 (p<0.005)	-	↑ 1.74 (p<0.05)	-	↑ 1.5 (p<0.001)
Hint-1	-	↑ 2.44 (p<0.0005)	-	-	↑ 1.8 (p<0.0005)
NDK I	-	-	↓ 3.45 (p<0.05)	-	↓ 1.6 (p<0.01)
40S ribosomal protein S12	-	-	-	-	↑ 2 (p<0.001)
SCRN1	-	↑ 13.6 (p<0.0005)	↑ 5.45 (p<0.01)	↑ 7.9 (p<0.00005)	↑ 3 (p<0.01)
Isoform ‘a’					
SCRN1	-	-	↓ 2.3 (p<0.005)	-	-
Isoform ‘b’					

(- indicates no significant change in expression)

**Table 3:** Average normalized volumes

**Age**	**Valosin containing protein**	**Secernin 1 (isoform a)**	**Secernin 1 (isoform b)**	**Beta Synuclein**	**Stathmin 1**	**Histidine triad nucleotide binding protein**	**Nucleoside diphosphate kinase**	**40S Ribosomal S12**
e12 wt and e12 *rdd*	0.019	0.06	0.24	0.14	0.004	0.47	0.314	0.605
e13 wt and e13 *rdd*	0.027	0.21	0.0004	0.0025	0.24	0.0004	0.114	0.355
e17 wt and e17 *rdd*	0.032	0.002	0.007	0.39	0.024	0.18	0.028	0.475
e19 wt and e19 *rdd*	0.5	0.02	0.000036	0.0005	0.48	0.37	0.33	0.218
p1 wt and p1 *rdd*	0.3	0.077	0.009	0.012	0.0008	0.0002	0.001	0.00095
One way anova wt	0.034	0.699	0.061	0.042	0.00013	0.011	0.000001	0.02
One way anova *rdd*	0.0087	0.00024	0.00014	0.0042	0.011	0.295	0.00000004	0.782

Probablity values determined using the Student’s *t*-test comparing the average normalized volumes of proteins identified by mass spectrometry from wt and *rdd* retina at each age, also shown are probability values for one way ANOVA (anova) for wt and *rdd* protein volume at each age.

**Figure 3:**
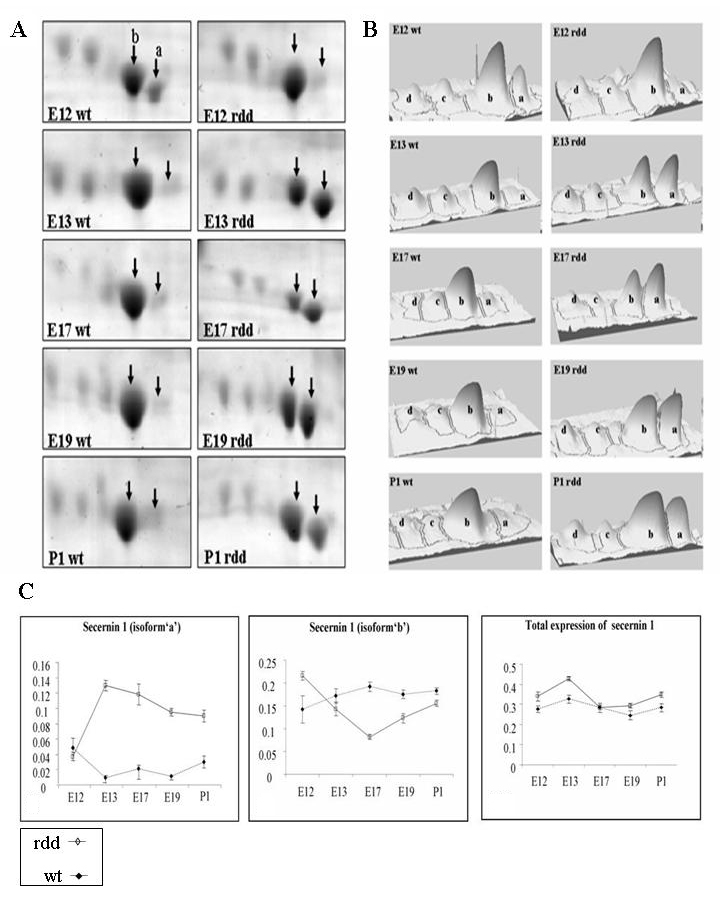
Differential expression of secernin 1 in the retinal dysplasia and degeneration (*rdd*) retina. **A**: Representative 2D montage images generated using Progenesis 2D image analysis software, revealing the modulated expression of secernin 1 in the wt and *rdd* chick. Two isoforms of secernin 1 (isoforms “a” and “b”) were identified by MS (arrows). The expression of isoform “a” is significantly increased from E13 onwards in the *rdd* retina, while it is only present at very low levels in the wt retina. **B**: 3D images of the 2D gels generated using Progenesis of the two isoforms of secernin 1 identified by MS. The increased expression of isoform “a” is evident from E13 onwards in the *rdd* retina. **C**: Graphical representation of expression of secernin 1 in the wt and *rdd* chick displaying the expression of isoform “a” (right panel), isoform “b” (middle panel), and total expression of secernin 1 (left panel). Age is plotted against average normalized volume (n=3±SEM).

The most significant outcome in this study was the differential expression of secernin 1 in the *rdd* retina. It appeared to resolve as four distinct spots (Figure 3A), two of which were identified by MS; resolution of secernin 1 along the acidic–basic axis is indicative of a posttranslational modification (PTM). Scansite Molecular Weight and Isoelectric Point Calculator analysis of the observed interspot pI distances (0.03 pI units) suggested that the observed interspot pI distances were compatible with differential phosphorylation states of secernin 1; however, this was not established definitively and would require further in depth MS analysis. Secernin 1 spots were present at all ages in the wt and *rdd* retina; however, there were significant differences between the expression of “isoform a” and “isoform b” (Figure 3C right panel and Figure 3C middle panel), with a 13.6 fold increase in the expression of “isoform a” in the *rdd* retina at E13 relative to wt expression at the same age. In fact, the expression of isoform “a” was significantly increased in the *rdd* retina up to P1 (Table 2). The total expression of all four isoforms of secernin 1 (Figure 3C left panel) was summed and compared using a Student *t*-test to determine if the total expression of secernin 1 in wt and *rdd* retina differed. This analysis revealed no significant difference in total expression at each age, thus indicating that the differences were due to PTM.

## Discussion

This is the first semiquantitative proteomic analysis of the developing and degenerating *rdd* retina, which presents with retinal degeneration akin to several severe forms of human retina degeneration. The mechanisms underlying retinal degeneration in RP remain poorly understood despite several decades of primarily genetic-driven research; these studies have exploited the increasing sophistication of genomic analysis of both human and animal models of retinal degeneration. Nonetheless, given the divergence of mutant genes identified, the progression of the underlying spectrum of pathologies is largely characterized by the general process of photoreceptor death, and yet these processes remain ill defined. The present study used histology to view the onset and progression of retinal degeneration, and proteomic analysis was used to identify proteins that were differentially expressed in the *rdd* retina.

Interestingly, four of the differentially expressed proteins identified in the *rdd* retina—valosin-containing protein, stathmin 1, 40S ribosomal protein S12, and nucleoside diphosphate kinase—have been shown to exhibit altered expression following proteomic analysis in animal models of the human neurodegenerative pathologies, Alzheimer and Parkinson diseases [23]. Valosin-containing protein is a member of the ubiquitously expressed multifunctional AAA+ (ATPase associated with various activities) protein family and has been implicated in a range of cellular functions, including organelle biogenesis and regulation of the ubiquitin–proteasome system [24]. Valosin-containing protein exhibited a lower parallel level of expression in the developing and early degenerate *rdd* versus the wt retina. Given the diversity of cellular roles attributed to valosin-containing protein, this observation in the *rdd* versus wt retina may reflect an overall decrease in cellular function.

Stathmin was detected in our previous report where it was shown to decrease significantly during normal retinal development in the chick [5]. In general, the pattern of stathmin expression observed in the developing *rdd* retina was more uniform with an overall decrease in expression at P1. Analysis of retinal regeneration in the newt *Cynops pyrrhogaster* detected increased stathmin expression from an early phase, and its expression continued until the retinal architecture was restored [25]. The observed stathmin modulation in the degenerate *rdd* retina versus the wt retina would be supportive of the concept that stathmin plays an important role in the construction and maintenance of retinal structure and its neural network [25].

Secernin 1 is a cystolic 50-kDa protein reported to regulate exocytosis in permeabilized mast cells [26]; however, its expression in the normal wt chick retina, bovine and rat brain, and in 12 normal human tissues with highest expression in nervous tissue would suggest a more generic function [26]. This view is supported by the ubiquitous expression of three secernin genes in many different murine [27] and human cells and is indicative of a widespread function rather than a specific role in mast cell exocytosis. This preliminary study has shown there is significant differential expression of secernin 1 in the *rdd* retina. Although there was no significant change in the total expression of secernin 1, there were significant differences in the expression of the different isoforms, namely isoform “a,” which was significantly upregulated at all ages in the *rdd* chick. Scansite Molecular Weight and Isoelectric Point Calculator analysis, which calculates the isoelectric point of a protein given different phosphorylation sites, predicted that these isoforms may represent phosphorylation events; however, this remains unsubstantiated and will require further analysis. Additionally, the different isoforms could be due to the expression of two or more secernin genes; two secernin genes have been identified in the chicken genome, secernin1 (located on chromosome 2) and secernin 2 (located on chromosome 7). The human genome contains three secernin genes, and there are at least two splice variants of secernin 2. Therefore different genes and splice variants may account for the four secernin 1 proteins identified in chick retina.

Given the widespread expression of secernin 1 in many tissues, secernin 1 is likely to have other functions beyond the regulation of mast cell exocytosis. Indeed, secernin 1 expression was upregulated in gastric cancer and colon cancer where it was implicated in cell growth [28], and it was found to be one of several novel genes overexpressed in Barrett's esophagus high-grade dysplasia [29]. Several proteomic studies have identified secernin 1 as being upregulated in the dorsolateral prefrontal cortex of patients with schizophrenia [30], and consistent with a decline in neuronal function, it was downregulated in the cerebral cortex of sleep-deprived mice [31]. Other studies suggest that secernin 1 is a neurotoxicologically relevant target as proteomic analysis of rat striatal synaptosomes identified secernin 1 as one protein that was progressively and significantly adducted during acrylamide intoxication [32]. At present, however, it is difficult to suggest a reasoned function for secernin within the retina and for its variant expression in the *rdd* retina, and as such, it is not possible to attribute any linkage between secernin 1 and Mupp1. Nevertheless, the data presented in this study clearly show a real alteration in secernin 1 expression in the degenerating retina that warrants further study. Preliminary analysis using the only secernin 1 antisera currently available detected numerous anomalous bands following western blot analysis, and while it did detect a cell population in the retina, these observations cannot be relied upon because of potential cross-reaction of antisera with unknown antigens expressed in retinal tissue.

The pathology displayed by the *rdd* chick resembles that observed in some of the more severe forms of human RP. It is now known that the *rdd* chicken carries a null mutation in the Mpdz/Mupp1 gene (Ali et al., manuscript in preparation). At present there are no reports in the literature of a direct association between mutations in Mpdz/Mupp1 and *rdd* in humans; there is, however, evidence to show that Mpdz/Mupp1is directly associated with Crumbs homolog 1 (Crb1). Crb1 is localized to the subapical region adjacent to the adherens junction complex at the outer limiting membrane in the retina, and loss of Crb1 function leads to either recessively inherited RP or LCA in humans [33]. This animal model may therefore offer valuable insights into the pathogenesis of RP, and the potential involvement of Mpdz/Mupp1 mutations in human inherited retinal disease is currently being investigated. The current proteomic analysis did not detect Mupp1, which has a molecular weight of approximately 220 kDa, not surprising given it is a large protein beyond the molecular-weight range arrayed in this study.

In summary, this investigation has successfully identified and provided novel data on several proteins differentially expressed during retinogenesis and with the onset of degeneration in the *rdd* chick. These proteins are involved in signaling, nucleoside biosynthesis, regulation of transcription, and protein synthesis. This study has generated novel data as some of these proteins have not previously been implicated in retinal degeneration. In particular this is the first report to demonstrate modulation of secernin 1 in relation to a retinal pathology. Consequently, the *rdd* chick would appear to be an important new animal model for the study of human RP.
